# *Tacca chantrieri* André Rhizome Extract Alleviates Scopolamine-Induced Cognitive Impairment and Neuroinflammation in Rats

**DOI:** 10.1155/adpp/7334303

**Published:** 2025-05-26

**Authors:** Thaneeya Hawiset, Napatr Sriraksa, Shisanupong Anukanon, Utcharaporn Kamsrijai, Siwaporn Praman, Narudol Teerapattarakan, Prachak Inkaew

**Affiliations:** ^1^School of Medicine, Mae Fah Luang University, Muang, Chiang Rai 57100, Thailand; ^2^School of Medical Sciences, University of Phayao, Muang, Phayao 56000, Thailand; ^3^School of Science, Mae Fah Luang University, Muang, Chiang Rai 57100, Thailand

**Keywords:** GFAP, memory, neuroinflammation, proinflammatory cytokine, scopolamine, serotonin, *Tacca chantrieri*

## Abstract

*Tacca chantrieri* André is a native plant from Northern Thailand with reported pharmacological effects, including antioxidant, anti-inflammatory, and neuroprotective properties. This study investigated the neuroinflammatory and cognitive-enhancing effects of *Tacca chantrieri* André rhizome extract (TCE) in a scopolamine-injected model, which mimics an Alzheimer's disease (AD) animal model. Animals were divided into six groups: (1) a control group, (2) a vehicle-treated group, (3) a donepezil-treated group (3 mg/kg BW) as a positive control, and (4–6) three TCE-treated groups receiving 50, 100, or 200 mg/kg BW once daily for 14 days. Starting on Day 8, animals received daily intraperitoneal injections of scopolamine (3 mg/kg BW) for 7 consecutive days to induce cognitive impairment. On day 14, behavioral tests were conducted, including the Y-maze and open field tests. On day 15, animals were euthanized, and their brains were collected for Nissl staining, immunofluorescence staining, and biochemical analyses using an ELISA kit. Our results demonstrated that TCE treatment attenuated scopolamine-induced memory deficits and neuroinflammation. Specifically, TCE administration reduced levels of proinflammatory cytokines, including tumor necrosis factor-α (TNF-α) and interleukin-1β (IL-1β), and decreased glial fibrillary acidic protein (GFAP) expression in the hippocampus. Additionally, TCE improved neuronal survival and enhanced serotonin levels, contributing to cognitive improvements. The qualitative analysis of TCE using LC-QTOF-MS identified various chemical constituents, including saponins, flavonoids, and phenolic compounds. These bioactive compounds contributed to the neuroprotective effects of TCE by modulating neuroinflammation and cognitive function. The neuroprotective effects of TCE suggested its potential as a therapeutic agent for memory impairment associated with AD.

## 1. Introduction

Alzheimer's disease (AD) is the most common neurodegenerative disorder, accounting for 60%–70% of dementia cases worldwide [1]. Neuroinflammation plays a key role in AD progression by triggering proinflammatory cytokine signaling, which disrupts neuronal function and contributes to cell death [2, 3]. Glial fibrillary acidic protein (GFAP), a key astrocytic cytoskeletal component, is released into the cerebrospinal fluid (CSF) during neuroinflammation and serves as a well-established marker of neurodegenerative diseases, including AD [4]. Additionally, peripheral inflammation involving cytokines such as tumor necrosis factor-α (TNF-α), interleukin-6 (IL-6), and interleukin-1β (IL-1β) could cross the blood–brain barrier (BBB), activating microglia and astrocytes and exacerbating neuronal damage [5].

Serotonin (5-hydroxytryptamine, 5-HT) is a neurotransmitter primarily associated with mood regulation, cognition, and neuroprotection [6]. Emerging evidence suggests that serotonin also plays a significant role in neuroinflammation and AD by modulating immune responses, reducing oxidative stress, and influencing amyloid-beta (Aβ) pathology [7, 8]. Serotonin regulates immune cell activity by interacting with microglia and astrocytes, facilitating the release of proinflammatory cytokines such as TNF-α, IL-1β, and IL-6 [9, 10]. Low serotonin levels contribute to overactive microglia, leading to sustained neuroinflammation and neuronal damage [10]. Additionally, serotonin modulates Aβ metabolism, and lower serotonin levels are correlated with increased Aβ aggregation [10]. Serotonin receptors, including 5-HT1A, 5-HT2A/C, and 5-HT4, exert neuroprotective effects by reducing tau hyperphosphorylation and amyloid-induced toxicity [9, 10]. In AD, serotonin levels decrease due to the degeneration of serotonergic neurons in the raphe nuclei of the brainstem. This decline impairs neurotransmission, negatively affecting cognition and mood [9, 10]. Given its role in reducing neuroinflammation, regulating Aβ metabolism, and protecting against tau pathology, targeting serotonin signaling pathways could offer a promising therapeutic approach for mitigating cognitive decline and neurodegeneration in AD.

Scopolamine-induced neuroinflammation is clinically relevant to AD pathophysiology, as it replicates key pathological features, including cholinergic dysfunction, neuroinflammation, oxidative stress, and cognitive impairment [11, 12]. By blocking muscarinic acetylcholine receptors (mAChRs), scopolamine disrupts cholinergic neurotransmission, leading to memory deficits similar to those observed in AD. Additionally, it activates microglia and astrocytes, increasing proinflammatory cytokines such as TNF-α, IL-1β, and IL-6, which contribute to neuronal damage and promote Aβ accumulation and tau hyperphosphorylation—hallmarks of AD [13, 14]. Scopolamine also induces oxidative stress by increasing reactive oxygen species (ROS) and causing mitochondrial dysfunction, leading to neuronal apoptosis and exacerbating neurodegeneration [15]. Furthermore, it impairs synaptic plasticity by reducing brain-derived neurotrophic factor (BDNF) levels, disrupting long-term potentiation (LTP), and accelerating cognitive decline [16, 17]. Because scopolamine effectively models early AD-like cognitive deficits and neuroinflammatory responses, it is widely used to evaluate potential AD therapies, including cholinesterase inhibitors, anti-inflammatory agents, antioxidants, and neuroprotective compounds. Given the increasing interest in alternative treatments, research into medicinal plants with neuroprotective properties remains essential. Exploring plant-based compounds that can counteract scopolamine-induced neuroinflammation may lead to promising therapeutic strategies for neurodegenerative disorders.

*Tacca chantrieri* André, a traditional medicinal plant native to Northern Thailand and belonging to the Dioscoreaceae family, was known for its rich phytochemical composition. Its rhizomes contained bioactive compounds such as saponins, glycosides, flavonoids, and phenolic compounds [18, 19]. These compounds contributed to the plant's diverse pharmacological properties, including antioxidant, neuroprotective, and anti-inflammatory activities [19–23]. Traditionally, *T. chantrieri* had been used in Thai medicine to treat ailments such as pain, fever, enteritis, and hepatitis [18, 20]. Recent research highlighted the therapeutic potential of *T. chantrieri* rhizome extract (TCE) in neurological disorders [22, 23]. Studies indicated that TCE possessed strong antioxidant activity [19, 21], which helped neutralize oxidative stress—one of the key contributors to neurodegeneration in AD. Additionally, TCE was shown to reduce neuroinflammation by inhibiting the production of proinflammatory cytokines such as TNF-α, which played a critical role in neuronal damage and cognitive decline [22]. In experimental models, TCE demonstrated protective effects against amyloid-beta peptide 25-35 (Aβ_25-35_)-induced neurotoxicity, a hallmark of AD pathology [23]. By mitigating oxidative stress and neuroinflammation, TCE helped preserve neuronal integrity and function. These neuroprotective effects suggested that TCE may have counteracted cognitive deficits and neuronal loss in AD-like conditions. Given these findings, this study aimed to further explore the neuroprotective effects of TCE in an AD animal model. Specifically, we investigated its potential to alleviate scopolamine-induced neuroinflammation and neuronal damage, providing new insights into its therapeutic potential for neurodegenerative diseases.

## 2. Materials and Methods

### 2.1. The Preparation of TCE From *T. chantrieri* André Rhizomes

Fresh rhizomes were obtained from Chiang Rai, Thailand, in September 2023 for the production of TCE. This plant was authenticated by Dr. Chaiyong Rujjanawate, and a voucher specimen (number 168-M) was consequently deposited at the School of Medicine, Mae Fah Luang University. The rhizomes were first thoroughly cleaned and dried. Then, they were ground into a coarse powder. Next, the powdered rhizome was extracted using 95% ethanol. The dry rhizome powder (4 kg) was extracted with 95% ethanol (20 L) at a 1:5 (w/v) ratio and repeated three times. Finally, the extract was filtered to remove any remaining solids. To concentrate the solution, the filtrate underwent evaporation. This concentration process was conducted utilizing a rotary evaporator under reduced pressure at temperatures ranging between 50 °C and 55 °C. The concentrated solution was subsequently subjected to freeze-drying to produce a solid powder. To preserve its stability and efficacy, this solid powder was kept in storage at −20 °C for further use.

### 2.2. Analysis of TCE by Liquid Chromatography-Quadrupole Time-of-Flight Mass Spectrometry (LC-QTOF-MS)

The extract of *Tacca chantrieri* André rhizomes were analyzed using Agilent Mass Hunter software, developed by Agilent Technologies Inc. The analysis was performed under the following conditions: a Poroshell EC-C18 column (2.7 μm, 2.1 × 150 mm), mobile phase A consisting of 0.1% formic acid in water, and mobile phase B containing 0.1% formic acid in acetonitrile (ACN). Additional parameters included a gas temperature of 300 °C, a gas flow rate of 10 L/min, a nebulizer pressure of 40 psi, a sheath gas flow rate of 12 L/min, a capillary voltage of 3500 V, and a nozzle voltage of 3.5 kV. The draw and ejection sampling speeds were set at 100 and 400 μL/min, respectively. Distilled water was used as the solvent to dilute the sample. A sample solution of 0.5 μL, with a concentration of 1 mg/mL, was injected for analysis. The flow rate was maintained at 0.2 mL/min. Mass spectra were acquired within the range of 50–1100 amu. The mass spectrometry (MS) scan rate was set at 4 spectra/s, with an equivalent MS/MS scan rate. Additionally, MS/MS analyses were performed in automatic mode, with collision energies of 10, 20, and 40 eV for fragmentation.

**Figure 1:**
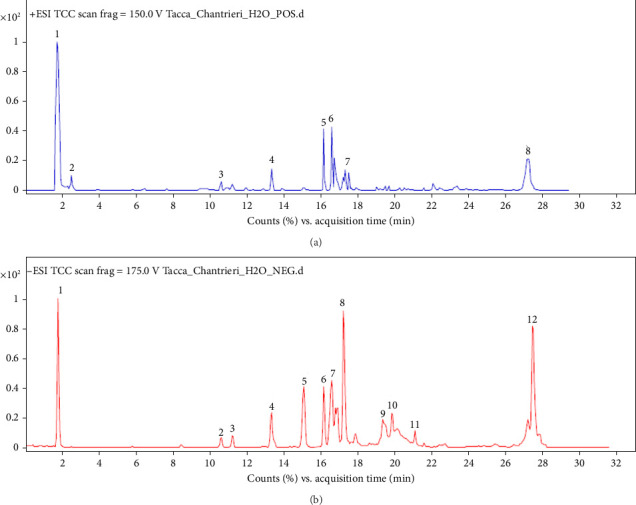
Total ion current chromatograms were monitored in (a) electrospray ionization positive mode and (b) negative mode.

The chromatograms of *Tacca chantrieri* André rhizome extract (TCE) obtained using LC-QTOF-MS/MS in both positive and negative modes are presented in Figures 1(a) and 1(b), respectively. The qualitative analysis of compounds in TCE identified significant chemical constituents, including saponins such as tragopogonsaponin G, basellasaponin A, kudzusaponin SA4, achyranthoside C, quillaic acid, ampeloside Bf2, and torvoside G. Additionally, the phenolic compounds and flavonoids identified included glucocaffeic acid, epiafzelechin, beta-hydroxyacteoside, 6-methoxyflavone, catechin 5-O-beta-D-glucopyranoside, quercetin 5,7,3′,4′-tetramethyl ether 3-rutinoside, 6,8-di-C-alpha-L-arabinopyranosylapigenin, ligustroside, and lucidenic acid B, as detailed in Table 1.

### 2.3. Animal Study

**Figure 2:**
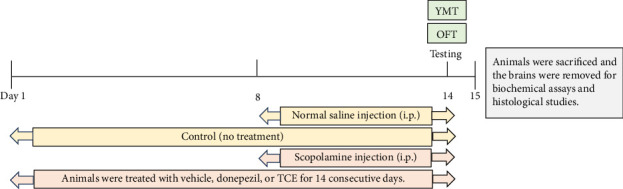
The protocol and design of the experiment. The animals were administered with vehicle, donepezil, or TCE (50, 100, or 200 mg/kg BW) once a day for 14 days. On day 8, scopolamine was intraperitoneally injected into the animals daily for the following 7 days. On day 14, the behavioral tests were investigated.

The study utilized 36 male Wistar rats, weighing between 220 and 240 g at 7 weeks of age, obtained from Nomura Siam International Co., Ltd. (Bangkok, Thailand). The sample size for the animal study was calculated using G∗Power 3.1.9.2 based on the sample size calculation from a previous study [24]. A stratified randomization protocol based on the body weight of animals was used in this study to ensure equal distribution of different weight groups across treatment and control groups. Each experimental group comprised six animals, with each cage housing three animals, and they were kept in a 12-h cycle of light and dark throughout the experiment's duration. Water and food were provided ad libitum to the animals. Before beginning the investigation, the animals were randomly assigned to various treatment groups. A 3-day acclimatization period was provided for the animals to familiarize themselves with the behavioral apparatus before undergoing evaluation. Behavioral tests were conducted on day 0 (baseline) and day 14. To ensure baseline equality, no significant differences in locomotor behaviors and cognitive behaviors were observed among the groups. On day 14, behavioral tests were conducted, which included the Y-maze test (YMT) and the open field test (OFT). After completing the behavioral tests, all animals were euthanized using an overdose of thiopental sodium. Due to the varying sensitivities of animals to anesthetic drugs, all animals received intraperitoneal injections of thiopental sodium at an initial dose of 70 mg/kg BW as a preliminary measure before increasing the dosage as necessary. Once the injectable anesthetic was administered, sufficient time was allowed for the animal to lose consciousness. If the animal did not lose consciousness, an additional anesthetic drug was administered. We confirmed that the animals exhibited no pain reflexes before sacrification. The final thiopental sodium dose used was approximately 70–100 mg/kg BW. Subsequently, their chest walls were opened, and blood was collected from the heart. Finally, the animals were euthanized immediately by removing the heart. To confirm animal death, the absence of a corneal reflex, failure to detect respiration, absence of a heartbeat, and no response to toe pinch were considered. The right hippocampal tissue was quickly removed for biochemical assays, while the left brains were obtained for histopathological studies. The study was conducted in accordance with internationally accepted principles for the use and care of laboratory animals, as outlined in the European Community Guidelines (EEC Directive of 1986; 86/609/EEC). The experiment was approved by the Laboratory Animal Research Center Ethics Committee of Mae Fah Luang University, with approval number AR03/66. The protocol and design of the experiment are outlined in Figure 2.

### 2.4. Drug Administration

The animals were separated into six groups, and the drugs were administered as follows: (1) control, (2) vehicle, (3) donepezil (DPZ) at a dosage of 3 mg/kg body weight (BW), (4) TCE at a dosage of 50 mg/kg BW, (5) TCE at a dosage of 100 mg/kg BW, and (6) TCE at a dosage of 200 mg/kg BW. The doses of DPZ and TCE were selected based on previously reported effective doses that demonstrate cognitive-enhancing effects in rodents [22, 25]. DPZ and TCE were dissolved in distilled water, while scopolamine was dissolved in 0.9% sodium chloride (normal saline). The animals were orally administered distilled water as a vehicle, DPZ (a cognitive enhancer drug manufactured by Eisai, Tokyo, Japan), or TCE once daily for 14 consecutive days. Each substance volume was of 1 mL/300 g of animal. On day 8, the control animals were intraperitoneally injected with 0.9% sodium chloride, while animals in other groups were intraperitoneally injected with scopolamine hydrobromide (SCO) at a concentration of 3 mg/kg BW (Sigma-Aldrich, USA) to induce cognitive impairment for the following 7 consecutive days [26].

### 2.5. The Behavioral Test of the Animals

#### 2.5.1. YMT

Spontaneous alternation behavior is assessed using the YMT to evaluate the cognitive ability of the animal [27]. Spontaneous alternation behavior serves as an investigation of the rats' exploratory behavior and their capacity to investigate new environments. Typically, animals tend to investigate the maze's arm that they have not previously explored. The animals were positioned at the center of the maze and given 5 minutes to freely investigate its three arms. The counting of arm entries was documented to evaluate the percentage of spontaneous alternation. When all four paws of an animal were contained within the arm of the apparatus, it was recorded as an arm entry. The percentage of spontaneous alternation behavior was determined using the formula [22]: Percentage of spontaneous alternations (%) = (Total number of alternations) × 100/(Total number of arm entries − 2)

#### 2.5.2. OFT

The OFT is a widely adopted method for evaluating rats' exploratory activities [28]. The apparatus employed for this test comprised a clear plexiglass box, divided into 16 equal squares. Each animal was positioned at the center of the field and permitted to freely investigate the box. The counting of crossings was determined by tallying the squares traversed by the animal with all four paws. The video camera recorded the activity for 5 minutes.

### 2.6. Biochemical Assessments

The right hippocampal tissue was promptly excised from the animals upon sacrifice and preserved at −80 °C for subsequent biochemical assays. Using a glass homogenizer, the tissue samples were weighed and homogenized with ice-cold phosphate-buffered saline (PBS). Following homogenization, the samples underwent centrifugation for 10 minutes at 10,000 × g at 4 °C to isolate the supernatant for further biochemical analyses. The supernatant was subsequently stored at −80 °C until further use.

A calibration curve was used in the biochemical analysis to quantify unknown samples. The obtained absorbance values of the samples were plugged into the calibration curve equation to determine their concentrations.

#### 2.6.1. Measurement of 5-HT Concentration

The concentration of 5-HT was determined using an enzyme-linked immunosorbent assay (ELISA) kit (Catalog number: E-EL-0033, Elabscience Biotechnology Co., Ltd, Houston, USA), following the manufacturer's instructions. Briefly, 50 μL of either the standard or sample was added to each well. Subsequently, 50 μL of biotinylated detection antibody was added to each well immediately and incubated for 45 minutes at 37 °C. The wells were then aspirated and washed three times. Next, 100 μL of HRP conjugate working solution was added to each well and incubated for 30 minutes at 37 °C. After aspirating and washing the wells three times, 90 μL of substrate reagent was added and incubated for 15 minutes at 37 °C. Finally, 50 μL of stop solution was added, and the absorbance was measured using a microplate reader at 450 nm. The 5-HT concentrations were normalized to protein content, and the experiments were conducted in duplicate.

#### 2.6.2. Measurement of TNF-α and IL-1β Levels

To assess the levels of proinflammatory cytokines such as TNF-α and IL-1β, an ELISA kit (Catalog number: E-EL-R2856; Catalog number: E-EL-R0012, Elabscience Biotechnology Co., Ltd., Houston, USA) was utilized in accordance with the manufacturer's instructions. In summary, 100 μL of the standard or sample was added to each well and incubated for 90 minutes at 37 °C. After this incubation period, the liquid was removed from the wells. Following this, 100 μL of biotinylated detection antibody working solution was introduced to each well and incubated for 60 minutes at 37 °C. The wells were then aspirated and washed three times. Subsequently, 100 μL of HRP conjugate working solution was added and incubated for 30 minutes at 37 °C. The wells were aspirated and washed five times. Afterward, 90 μL of substrate reagent was added and incubated for 15 minutes at 37 °C. Lastly, 50 μL of stop solution was added, and the absorbance was measured using a microplate reader at 450 nm. The experiments were conducted in duplicate.

#### 2.6.3. Measurement of Protein Concentration

The total protein concentration of the whole-cell lysate was determined using the colorimetric Bradford technique and a commercial protein assay kit (Catalog number: SK3031, Ontario, Canada). For calibration, bovine serum albumin (BSA) was used as a standard reference. The range of BSA standards used to ensure the accuracy of protein concentration measurements was 0, 10, 20, 30, 40, 60, 80, 100, and 125 μg/mL. The results were obtained in triplicate.

### 2.7. Tissue Processing and Nissl Staining

The left brains were quickly removed and immersed in 4% paraformaldehyde in 0.1 M PBS at pH 7.4. Subsequently, the brains were preserved in paraffin and sectioned serially at a thickness of 5 μm using a Leica rotatory microtome (RM 2245 model, Leica, Wetzlar, Germany). After sequentially immersing the brain slices in xylene, ethanol, and distilled water for 5 minutes each, they were stained with 1% cresyl violet (Sigma-Aldrich, USA). Then, they were rinsed twice with distilled water and sequentially dehydrated for 5 minutes each in ethanol concentrations ranging from 70% to 100%. The slides were subsequently cleared three times with xylene and finally mounted with a mounting medium (Sigma-Aldrich, USA).

Images of the hippocampal region were captured at a magnification of 20x using an Olympus EP50 microscope equipped with the EP view program (Olympus Corporation, Tokyo, Japan). Neurons in the dentate gyrus (DG) area were counted by a researcher who was unaware of the group of animals from which the stained neurons were obtained. The density of survival neurons was then determined. After reviewing the images at 20x magnification, a fixed area of 200 μm^2^ was selected. Neuron density was determined by counting the neurons within this region, and the results were reported as neuron density (cells/200 μm^2^). To evaluate Nissl staining, three photomicrographs were used per defined region for each animal.

### 2.8. Immunofluorescence Staining

The method begins with the deparaffinization process, which involves sequentially immersing the samples in xylene three times, followed by immersion in ethanol at concentrations of 95%, 90%, 80%, and 70%. Antigen retrieval was performed by immersing 0.2 M Tris–HCl, followed by autoclaving at 95 °C for 15 minutes. After cooling to room temperature, the slides were washed twice with PBS. Subsequently, 0.1% Triton X-100 was applied for 15 minutes, followed by an additional PBS wash. The slides were then treated with a blocking solution consisting of 5% BSA in PBS for 30 minutes, followed by another PBS wash. The slides were incubated with the primary antibody, GFAP monoclonal antibody (1:200 dilution; MA512023, Thermo Fisher Scientific, U.S.A.), overnight at 4 °C. Subsequently, the secondary antibody, goat anti-mouse IgG H&L conjugated to Alexa Fluor 594 (1:300 dilution; AB150116, Abcam, Cambridge, UK), was applied for 1 hour at room temperature. After another PBS wash, Hoechst 33528 (blue; 1:2000 dilution, AB228550, N marker, Abcam, Cambridge, UK) was added for 10 minutes and used immediately. Finally, the slides were washed with PBS for 3 minutes each before applying an anti-fade fluorescence mounting medium (AB104135, Abcam, Cambridge, UK) and sealing the slide edges with nail polish. The immunohistochemical reaction was observed under a Zeiss Axio Scope A1 microscope (Carl Zeiss Suzhou Co., Ltd., Suzhou, China).

In this investigation, the percentage of GFAP expression was determined in the DG region of the rat brain. Three brain samples from each group were randomly selected, and five sections per sample were analyzed. Digital images of 40 pictures per group were captured at a magnification of 20x. Subsequently, GFAP expression data were quantified using ImageJ analysis.

### 2.9. Statistical Analysis

The statistical analysis was conducted using SPSS Statistics 25 software, and the results were presented as mean ± standard error of the mean (SEM). To evaluate significant differences among means of multiple groups, one-way ANOVA was employed, followed by Tukey's honestly significant difference (HSD) post hoc test. Statistical assumptions were verified using Levene's test for homogeneity of variance. A statistical significance level of *p* < 0.05 was applied to determine the significance of the results.

## 3. Results

### 3.1. Effect of TCE on Scopolamine-Induced Memory Deficit and Locomotor Behavior

**Figure 3:**
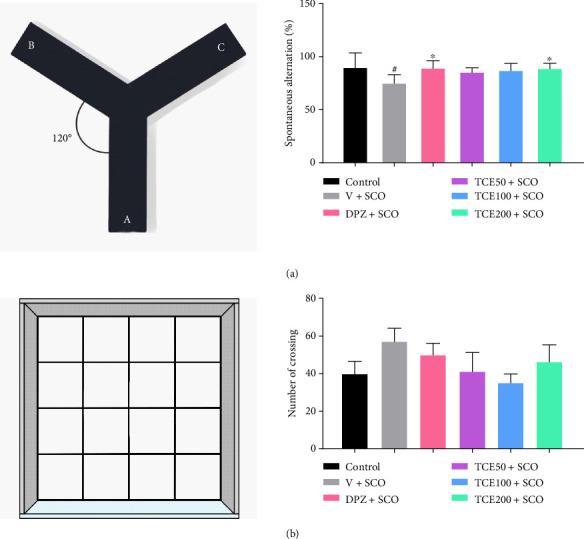
Effect of TCE on scopolamine-induced memory deficit and locomotor behavior. (a) Diagram of the Y-maze test (left) and a graph showing the results of spontaneous alternation behavior (right). (b) Diagram of open field test (left) and a graph showing the results of the number of crossings (right). One-way ANOVA was used to analyze the data, and the mean ± SEM (*n* = 6) was presented as the outcome. ^#^*p* < 0.05 versus the control group. ^∗^*p* < 0.05 versus the vehicle with the scopolamine group.

The YMT is frequently used in AD research because dysfunction in the hippocampus and prefrontal cortex—key regions affected in AD—impairs spontaneous alternation performance, a measure of spatial working memory and cognitive flexibility [27]. Our result showed that memory impairments were observed in the animals injected with scopolamine, as indicated by a decreased proportion of spontaneous alternation behavior (F_(4,25)_ = 1.465, *p* < 0.05) compared to the control group. However, animals treated with DPZ and TCE (200 mg/kg BW) and subsequently injected with scopolamine exhibited significantly improved cognitive performance compared to the untreated group that also received scopolamine (*p* < 0.05 for both treatment groups), as shown in Figure 3(a). These results confirmed that scopolamine-induced cognitive impairment reduced alternation behavior, mimicking memory deficits observed in AD. The improvement in alternation behavior following TCE treatment suggested its potential to enhance cognitive function.

The OFT is a widely used behavioral test to assess locomotor activity and exploratory tendencies in animals, particularly rodents [28]. In Figure 3(b), when the OFT was used to assess exploration behavior, the scopolamine-injected animals treated with vehicle, DPZ, and TCE at all dosages used in this study did not exhibit a significantly different number of crossings compared to the control group (F_(4,25)_ = 0.817). These findings suggest that scopolamine did not affect locomotor behavior. Our study utilized a low dose of scopolamine, which gradually impaired cognitive performance similar to that observed in AD patients. As a result, locomotor activity remained unchanged between groups.

### 3.2. Effect of TCE on Proinflammatory Cytokine Levels and 5-HT Concentration

**Figure 4:**
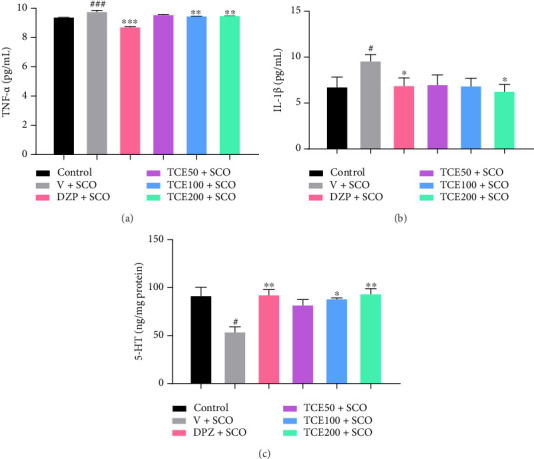
Graphs of the effect of TCE on (a) TNF-α levels, (b) IL-1β levels, and (c) 5-HT concentration. One-way ANOVA was used to analyze the data, and the mean ± SEM (*n* = 6) was presented as the outcome. ^#^*p* < 0.05 and ^###^*p* < 0.001 versus the control group; ^∗^*p* < 0.05^∗∗^*p* < 0.01, and ^∗∗∗^*p* < 0.001 versus the vehicle with the scopolamine group.

5-HT plays a crucial role in modulating neuroinflammation by regulating the production and release of proinflammatory cytokines, including TNF-α and IL-1β. It interacted with immune cells, including microglia and astrocytes, to influence inflammatory responses in the central nervous system (CNS) [7, 9]. TNF-α is a proinflammatory cytokine that plays a pivotal role in initiating and amplifying neuroinflammatory processes. It contributes to neuronal dysfunction by promoting oxidative stress, BBB disruption, and apoptosis. Elevated TNF-α levels are commonly observed in AD [3]. Furthermore, IL-1β is another major proinflammatory cytokine that regulates immune responses and mediates neuroinflammation. It enhances the production of other inflammatory mediators, disrupts synaptic plasticity, and is linked to cognitive impairments in neurodegenerative disorders [3].  Figure 4(a) illustrates that animals administered a vehicle along with a scopolamine injection exhibited higher levels of TNF-α (F_(4,25)_ = 46.403, *p* < 0.001) and IL-1β (F_(4,25)_ = 1.525, *p* < 0.05) than the control group. In contrast, TNF-α levels of animals treated with DPZ, TCE (100 and 200 mg/kg BW), and scopolamine injections were lower than those of animals administered a vehicle (*p* < 0.001, *p* < 0.01, *p* < 0.01, respectively). Furthermore, animals injected with scopolamine and treated with DPZ and TCE (200 mg/kg BW) exhibited lower IL-1β levels compared to the untreated group receiving scopolamine (*p* < 0.05 for both groups), as depicted in Figure 4(b).

The data presented in Figure 4(c) indicate that animals receiving a vehicle and scopolamine injections expressed lower levels of 5-HT (F_(4,25)_ = 2.190, *p* < 0.05) compared to the control animals. Additionally, animals treated with DPZ and TCE (100 and 200 mg/kg BW) expressed higher levels of 5-HT (*p* < 0.01, *p* < 0.05, and *p* < 0.01, respectively) than animals that received vehicle and scopolamine. Our results demonstrated that scopolamine-induced cognitive impairment and neuroinflammation occur through the disruption of serotonergic pathways. The resulting decrease in serotonin levels led to an increase in proinflammatory cytokines, creating a vicious cycle of inflammation and neuronal damage. Interestingly, DPZ and TCE treatments enhanced serotonin signaling and reduced TNF-α and IL-1β levels in the brain. These findings suggest that TCE may exert neuroprotective effects and contribute to memory improvement in an AD animal model.

### 3.3. Effect of TCE on Neuronal Survival in the Hippocampus

**Figure 5:**
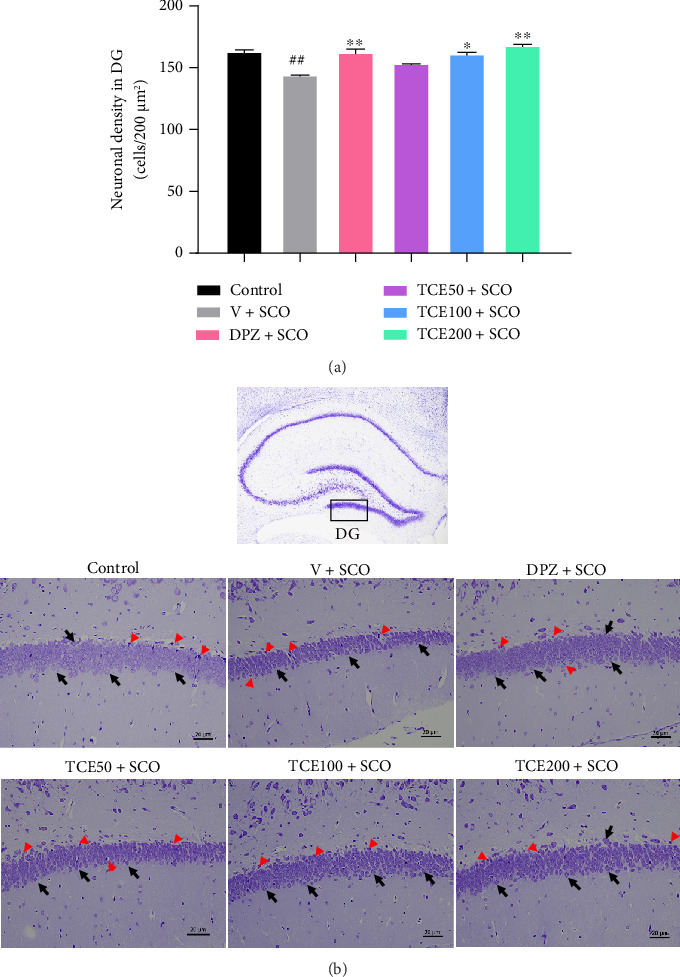
Effect of TCE on neuronal density in the hippocampus. (a) Graph of neuronal density in the DG region of the hippocampus. (b) Photomicrographs of the DG sections of the hippocampus at a 20x magnification. Scale bars with a length of 20 μm accompany these images. The red arrowhead indicated the dark, shrunken, and damaged neurons, while the black arrow indicated the neurons that were still alive. One-way ANOVA was used to analyze the data, and the mean ± SEM (*n* = 6) was presented as the outcome. ^##^*p* < 0.01 versus the control group; ^∗^*p* < 0.05 and ^∗∗^*p* < 0.01 versus the vehicle with the scopolamine group.

The hippocampus is a critical brain region involved in learning and memory, and its dysfunction is a hallmark of AD. Neuronal survival in the hippocampus is essential for maintaining cognitive function, and its decline is closely linked to disease progression [29]. The neuronal density of the DG is presented in Figure 5(a). Compared to the control animals, animals administered a vehicle and scopolamine exhibited a significantly lower number of neuronal survival in the DG region (F_(4,25)_ = 6.286, *p* < 0.01). In contrast to animals administered a vehicle, animals receiving scopolamine injection and treated with DPZ and TCE (100 and 200 mg/kg BW) demonstrated higher neuronal survival in the hippocampal DG area (*p* < 0.01, *p* < 0.05, and *p* < 0.01, respectively). Our findings showed that scopolamine effectively induced neuronal loss in the hippocampus by activating astrocytes to release proinflammatory cytokines (e.g., TNF-α and IL-1β), exacerbating neuronal damage. However, TCE treatment demonstrated neuroprotective properties by reducing proinflammatory cytokines, enhancing serotonin levels to modulate inflammation, and protecting hippocampal neurons from scopolamine-induced neuronal damage. Nissl staining was utilized to capture photomicrographs of the DG sections of the hippocampus at a magnification of 20x, as demonstrated in Figure 5(b).

### 3.4. Effect of TCE on GFAP Expression in the Hippocampus

**Figure 6:**
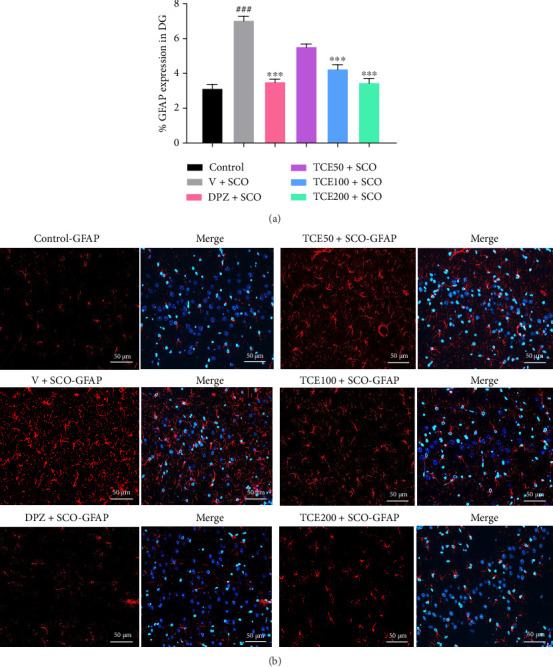
Effect of TCE on the percentage of GFAP expression in the hippocampus. (a) Percentage of GFAP expression in the DG. (b) The immunofluorescence of GFAP (red) expression in the DG, Hoechst 33258 (blue), and merged images at a 20x magnification. Scale bars measuring 50 μm accompany these images. One-way ANOVA was used to analyze the data, and the mean ± SEM (*n* = 6) was presented as the outcome. ^###^*p* < 0.001 versus the control group; ^∗∗∗^*p* < 0.001 versus the vehicle with scopolamine group.

GFAP is an intermediate filament protein primarily expressed in astrocytes, the star-shaped glial cells responsible for maintaining neuronal homeostasis. GFAP plays a crucial role in astrocyte structure, function, and response to brain injuries, including neuroinflammation and neurodegeneration [30]. The animals injected with scopolamine and administered with a vehicle exhibited a higher percentage of GFAP expression in the hippocampal DG region (F_(4,25)_ = 67.899, *p* < 0.001) compared to the control animals, as shown in Figure 6(a). However, treatment with DPZ and TCE (100 and 200 mg/kg BW) demonstrated a lower percentage of GFAP expression in the hippocampal DG region (*p* < 0.001 for all groups). Scopolamine-injected animals, which mimicked AD pathology, exhibited reactive astrocytes in response to neuroinflammation. This led to an increase in GFAP expression, a well-established marker of astrocyte activation (astrogliosis). However, the observed reduction in the GFAP expression following TCE treatment suggested decreased astrocytic reactivity, indicating a neuroprotective effect in mitigating neuroinflammation.

Since GFAP is a key marker of astrocyte activation in response to neuronal injury or inflammation, its downregulation might indicate a reduction in astrocyte-mediated inflammatory response. This decrease in astroglial activation could create a neuroprotective environment, helping to preserve neuronal integrity and function against scopolamine-induced neurotoxicity. These findings suggested that TCE might counteract astrocyte-driven neuroinflammation, supporting cognitive function and neuronal survival in animal AD models.  Figure 6(b) represents the immunofluorescence staining of GFAP expression in the hippocampal DG region at a magnification of 20x.

## 4. Discussion

This study demonstrated that TCE effectively mitigated scopolamine-induced cognitive impairment and neuroinflammation through multiple mechanisms. Specifically, TCE reduced proinflammatory cytokines (TNF-α and IL-1β), downregulated GFAP expression—indicating decreased astrocyte reactivity—and enhanced serotonin levels, which might contribute to improved synaptic function and neuroprotection. These findings suggested that TCE exerted neuroprotective effects by modulating both inflammatory and neurotransmitter pathways, ultimately supporting cognitive function. The following discussion elaborates on these mechanisms and their implications for neurodegenerative conditions.

Neuroinflammation is a key factor in the development of AD, the most common form of dementia [2, 3]. Growing evidence suggests that persistent neuroinflammation, primarily mediated by glial cells, drives both neurodegeneration and cognitive impairment in AD [30, 31]. Neuroinflammation refers to an inflammatory response within the CNS triggered by pathological damage, either within the CNS itself or from peripheral sources. This response leads to the production of proinflammatory cytokines such as IL-1β, IL-6, IL-8, and TNF-α [32, 33]. Scopolamine negatively impacts neuronal development and disrupts hippocampal circuitry, particularly in the hippocampal DG region. It acts as a neurotoxin within the CNS, leading to cognitive impairment [34, 35]. Its administration induces brain inflammation, which in turn increases inflammatory cytokine levels [13]. Our study found that animals receiving scopolamine and treated with TCE exhibited reduced levels of proinflammatory cytokines, including TNF-α and IL-1β. These results align with previous research showing that TCE mitigates neuroinflammation and decreases TNF-α levels in LPS-induced models [22]. This suggests that TCE has a protective effect against neuroinflammation.

Astrocyte activation contributes to neuroinflammation and plays a key role in neurodegeneration [36, 37]. Evidence suggests that activated astrocytes produce higher levels of GFAP, a key cytoskeletal protein [37]. GFAP also plays a crucial role in synaptic function and astrocyte–neuron interactions within the CNS [38]. Additionally, research indicates that regulating astrocyte activity can influence neuronal survival and cognitive function [39]. Neuroinflammation and astrocyte activation are pathological factors that affect hippocampal tissues [29, 34]. The DG plays a crucial role in neurogenesis by mediating the proliferation of neural stem cells, which differentiate into neurons and astrocytes [34]. Activated astrocytes typically express increased GFAP and undergo hypertrophic morphological changes [40]. This study focuses on the protective effects of TCE, particularly in preserving neuronal survival and modulating astrocyte activation to support cognitive function. Our findings showed that scopolamine significantly reduced neuronal density in the hippocampal DG compared to controls. However, TCE treatment effectively prevented neuronal loss in this region. Additionally, scopolamine increased GFAP expression in the DG, indicating astrocyte activation. In contrast, animals treated with TCE at 100 and 200 mg/kg BW exhibited a reduction in the GFAP expression, suggesting a protective effect against scopolamine-induced astrocyte activation. TCE's reduction of GFAP expression may have significant implications for synaptic function and astrocyte–neuron interactions beyond merely mitigating inflammation.

Astrocytes play a crucial role in synaptic homeostasis by regulating neurotransmitter uptake, ion balance, and the release of gliotransmitters that modulate neuronal activity [41]. A decrease in GFAP expression could reflect a shift from a reactive to a more homeostatic state, potentially enhancing astrocytic support for synapses. This may improve glutamate clearance, reduce excitotoxicity, and promote synaptic plasticity, thereby supporting a neuroprotective environment [42]. Furthermore, astrocytes provide metabolic support by supplying neurons with lactate and reduced GFAP expression might suggest improved metabolic coupling, enhancing neuronal plasticity [41, 42]. A previous study identifies a link between elevated GFAP expression and the overproduction of proinflammatory cytokines, including TNF-α and IL-1β [43]. These cytokines play a key role in disorders associated with acute neuroinflammation [44] and are primarily produced by microglia and astrocytes [45, 46]. Our results showed that scopolamine injection significantly increased TNF-α and IL-1β expressions in the hippocampus, indicating heightened neuroinflammation. However, TCE treatment at 200 mg/kg BW effectively reduced the expression of these cytokines in hippocampal tissues. Additionally, TCE enhanced neuronal survival by suppressing GFAP, TNF-α, and IL-1β expressions. These findings aligned with previous studies demonstrating that TCE mitigated memory impairment and neuroinflammation by reducing TNF-α levels in the brain [22] and protected neuronal cells from Aβ_25-35_-induced damage [23].

Serotonin plays a crucial role in synaptic plasticity, learning, and memory by modulating neurotransmission in key brain regions such as the hippocampus and prefrontal cortex [9, 47]. Elevated serotonin levels enhance LTP, essential for memory formation, by strengthening excitatory synapses [48]. Additionally, serotonin regulates neurogenesis and dendritic spine density, both critical for cognitive resilience and adaptability [49]. Another key mechanism involves serotonin's role in reducing oxidative stress and neuroinflammation, both of which are exacerbated by scopolamine-induced cognitive impairment. By lowering inflammatory cytokine release and promoting neuronal survival, serotonin counteracts scopolamine's neurotoxic effects [50]. These mechanisms suggest that TCE's cognitive benefits extend beyond reducing inflammation and directly enhancing serotonergic function to support learning and memory. Furthermore, our findings showed that scopolamine effectively induced cognitive impairment in animals, as evidenced by a decrease in YMT-based spontaneous alternation behavior. Administering TCE to scopolamine-treated animals resulted in improved cognitive performance. These observations were consistent with increased brain serotonin levels, which have been associated with enhanced cognitive function [48]. Considering all findings, TCE treatment mitigated scopolamine-induced memory impairment by reducing proinflammatory cytokines and GFAP expression while concurrently increasing 5-HT concentration and neuronal density in the hippocampus.

The dose of DPZ at 3 mg/kg BW was directly compared with the administered TCE doses of 50, 100, and 200 mg/kg BW to provide a clearer understanding of their relative efficacy. DPZ, a cholinesterase inhibitor, was well documented at this dosage for reversing scopolamine-induced cognitive impairment, primarily by enhancing cholinergic neurotransmission. A dose–response comparison between DPZ and TCE helped determine whether TCE exhibited neuroprotective effects comparable to DPZ and whether its efficacy increased with dosage. Our findings reported that TCE at higher doses (200 mg/kg BW) showed similar or superior cognitive improvement to DPZ, suggesting a potential therapeutic benefit, possibly through additional mechanisms such as serotonergic modulation and anti-inflammatory activity. Additionally, examining whether TCE's effects plateaued or continued to increase with dose escalation could provide insights into its optimal therapeutic range compared to DPZ's well-established efficacy at 3 mg/kg BW.

**Figure 7:**
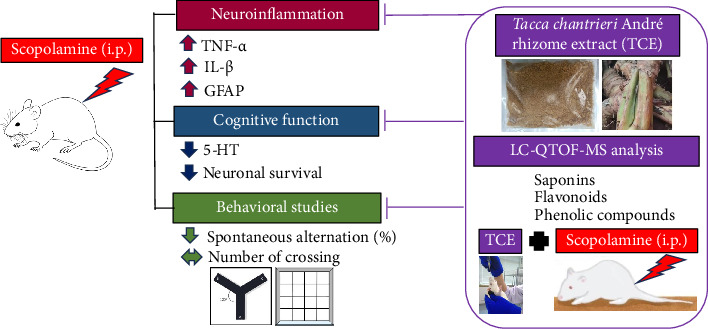
A schematic diagram illustrates the possible mechanisms TCE protects against scopolamine-induced memory impairment and neuroinflammation.

TCE demonstrated efficacy in mitigating the effects of scopolamine, likely due to the action of its bioactive ingredients, including saponins, catechin, quercetin, flavones, epiafzelechin, and lucidenic acid B (Table 1). Each of these compounds modulated key inflammatory and neuroprotective pathways relevant to scopolamine-induced impairments. Saponins reduced proinflammatory cytokines such as TNF-α, IL-1β, and IL-6 [51] while also enhancing hippocampal 5-HT and noradrenaline levels [51, 52], which aligned with the observed reductions in neuroinflammation and improvements in memory function. Catechin and quercetin similarly attenuated neuroinflammatory responses by decreasing TNF-α and IL-1β [53–55], supporting the reductions in inflammatory markers seen in our results. Flavonoids helped preserve neuronal and astrocyte morphology [56], consistent with the neuroprotective effects observed in our histological analysis. Epiafzelechin protected against β-amyloid-induced neuronal death [57], which may have contributed to the preservation of neuronal integrity seen in our study. Lastly, lucidenic acid B suppressed inflammatory cytokine secretion, reinforcing the reduction in neuroinflammatory markers [58]. Collectively, these compounds counteracted scopolamine's neurotoxic effects, as illustrated in Figure 7, which summarized the potential mechanisms underlying TCE's neuroprotective action.

To address the overlooked neuroinflammatory pathways, it would be beneficial to discuss key mediators such as IL-6 and iNOS, which play significant roles in AD pathology. IL-6 is a proinflammatory cytokine that contributes to neuroinflammation, synaptic dysfunction, and tau hyperphosphorylation, making it a crucial target for neuroprotective interventions [59]. Similarly, iNOS is involved in oxidative stress and neurotoxicity through excessive nitric oxide production, which can exacerbate neuronal damage [60]. Exploring whether TCE modulates these pathways would provide a more comprehensive view of its neuroprotective potential. Additionally, the use of a male-only cohort overlooks potential sex differences in neuroinflammation and serotonin modulation. Females often exhibit heightened neuroinflammatory responses due to hormonal influences on cytokine regulation, which could alter the effects of TCE on glial activation and inflammatory signaling. Likewise, serotonergic function differs between sexes, potentially affecting how TCE enhances cognitive performance. Future studies incorporating both sexes would improve the generalizability of findings and provide insights into sex-specific therapeutic effects of TCE in neurodegenerative conditions. The cholinergic and glutamatergic neurotransmitter systems are essential for cognition, learning, and memory. Dysfunction in these pathways is strongly linked to AD, contributing to cognitive decline and neurodegeneration. The lack of evaluation of these systems is a limitation of this study, which future research should address. While scopolamine is primarily used to model cognitive deficits by blocking cholinergic activity, it also triggers neuroinflammatory responses similar to those in AD, including increased cytokine release and astrocyte activation. These inflammatory changes, along with oxidative stress and synaptic dysfunction, mimic early-stage AD, making scopolamine a useful but short-term model for testing neuroprotective treatments. Further investigation into how TCE's effects translate to chronic neurodegenerative conditions could enhance its clinical relevance.

## 5. Conclusion

Our results demonstrated that TCE exhibited neuroprotective effects against scopolamine-induced neuronal loss and neuroinflammation by reducing proinflammatory cytokines and GFAP expression. This reduction led to enhanced cognitive function and improved neuronal density. Consequently, TCE showed potential for treating AD by mitigating neuroinflammation-induced neuronal loss. The presence of various bioactive components in TCE, including saponins, quercetin, catechin, flavones, epiafzelechin, and lucidenic acid B, likely contributed to its neuroprotective effects. These results could have been due to the synergistic action of multiple compounds against neuroinflammation. However, further studies were needed to determine the optimal therapeutic dose, assess its bioavailability, and explore lower doses that might still provide significant pharmacological effects. Future research should also focus on identifying the active compounds responsible for TCE's neuroprotective properties.

## Figures and Tables

**Table 1:** Qualitative compound identification of *Tacca chantrieri* André rhizome extract was determined by LC-ESI-QTOF-MS/MS analysis.

Peak no.	Proposed compound	RT (min)	Molecular weight	Formula
*LC-ESI-QTOF-MS/MS (positive mode)*				
1	Glucocaffeic acid	1.76	342.10	C_15_H_18_O_9_
2	Epiafzelechin (2R,3R) (−)	2.28	274.08	C_15_H_14_O_5_
3	Eudesmin	10.19	386.17	C_22_H_26_O_6_
4	Catechin 5-O-beta-D-glucopyranoside	13.38	452.13	C_21_H_24_O_11_
5	Quercetin 5,7,3′,4′-tetramethyl ether 3-rutinoside	16.13	666.22	C_31_H_38_O_16_
6	6,8-Di-C-alpha-L-arabinopyranosylapigenin	16.75	534.13	C_25_H_26_O_13_
7	Beta-hydroxyacteoside	17.57	640.20	C_29_H_36_O_16_
8	N-palmitoyl tryptophan	27.34	442.32	C_27_H_42_N_2_O_3_

*LC-ESI-QTOF-MS/MS (negative mode)*				
1	Glucocaffeic acid	1.76	342.10	C_15_H_18_O_9_
2	Tragopogonsaponin G	10.21	1090.53	C_56_H_82_O_21_
3	Basellasaponin A	11.29	970.44	C_47_H_70_O_21_
4	Kudzusaponin SA4	13.21	958.48	C_47_H_74_O_20_
5	6-Methoxyflavone	15.33	252.08	C_16_H_12_O_3_
6	Nigericin	16.60	724.48	C_40_H_68_O_11_
7	Ligustroside	16.90	524.19	C_25_H_32_O_12_
8	Achyranthoside C	17.29	956.46	C_47_H_72_O_20_
9	Quillaic acid	19.68	486.70	C_30_H_46_O_5_
10	Ampeloside Bf2	20.08	952.49	C_45_H_76_O_21_
11	Torvoside G	21.18	608.29	C_34_H_56_O_9_
12	Lucidenic acid B	27.78	474.26	C_27_H_38_O_7_

*Note:* min, minute.

Abbreviations: ESI, electrospray ionization; RT, retention time.

## Data Availability

The data that support the findings of this study are available from the corresponding author upon reasonable request.

## References

[1] Revi M. (2020). Alzheimer’s Disease Therapeutic Approaches. *Advances in Experimental Medicine and Biology*.

[2] Kwon H. S., Koh S. H. (2020). Neuroinflammation in Neurodegenerative Disorders: The Roles of Microglia and Astrocytes. *Translational Neurodegeneration*.

[3] Wang W. Y., Tan M. S., Yu J. T., Tan L. (2015). Role of Pro-Inflammatory Cytokines Released From Microglia in Alzheimer’s Disease. *Annals of Translational Medicine*.

[4] Yang Z., Wang K. K. (2015). Glial Fibrillary Acidic Protein: From Intermediate Filament Assembly and Gliosis to Neurobiomarker. *Trends in Neurosciences*.

[5] Winkler B., Funke D., Benmimoun B. (2021). Brain Inflammation Triggers Macrophage Invasion across the Blood-Brain Barrier in *Drosophila* during Pupal Stages. *Science Advances*.

[6] Ciranna L. (2006). Serotonin as a Modulator of Glutamate-and GABA-Mediated Neurotransmission: Implications in Physiological Functions and in Pathology. *Current Neuropharmacology*.

[7] Metaxas A., Anzalone M., Vaitheeswaran R., Petersen S., Landau A. M., Finsen B. (2019). Neuroinflammation and Amyloid-Beta 40 Are Associated with Reduced Serotonin Transporter (SERT) Activity in a Transgenic Model of Familial Alzheimer’s Disease. *Alzheimer’s Research & Therapy*.

[8] He M., Park C., Shin Y., Kim J., Cho E. (2023). N-feruloyl Serotonin Attenuates Neuronal Oxidative Stress and Apoptosis in Aβ_25–35_-Treated Human Neuroblastoma SH-Sy5y Cells. *Molecules*.

[9] Afshar S., Shahidi S., Rohani A. H., Soleimani Asl S., Komaki A. (2019). Protective Effects of 5-HT1A Receptor Antagonist and 5-HT2A Receptor Agonist on the Biochemical and Histological Features in a Rat Model of Alzheimer’s Disease. *Journal of Chemical Neuroanatomy*.

[10] Madsen K., Neumann W. J., Holst K. (2011). Cerebral Serotonin 4 Receptors and Amyloid-β in Early Alzheimer’s Disease. *Journal of Alzheimer’s Disease*.

[11] Hawiset T., Sriraksa N., Kamsrijai U., Praman S., Inkaew P. (2023). Neuroprotective Effect of *Tiliacora Triandra* (Colebr.) Diels Leaf Extract on Scopolamine-Induced Memory Impairment in Rats. *Heliyon*.

[12] Chen W. N., Yeong K. Y. (2020). Scopolamine, a Toxin-Induced Experimental Model, Used for Research in Alzheimer’s Disease. *CNS & Neurological Disorders-Drug Targets*.

[13] Cheon S. Y., Koo B. N., Kim S. Y., Kam E. H., Nam J., Kim E. J. (2021). Scopolamine Promotes Neuroinflammation and Delirium-like Neuropsychiatric Disorder in Mice. *Scientific Reports*.

[14] Jo S. H., Kang T. B., Koppula S. (2021). Mitigating Effect of *Lindera Obtusiloba* Blume Extract on Neuroinflammation in Microglial Cells and Scopolamine-Induced Amnesia in Mice. *Molecules*.

[15] Salimi A., Sabur M., Dadkhah M., Shabani M. (2022). Inhibition of Scopolamine-Induced Memory and Mitochondrial Impairment by Betanin. *Journal of Biochemical and Molecular Toxicology*.

[16] Yu H., Lv D., Shen M. (2019). BDNF Mediates the Protective Effects of Scopolamine in Reserpine-Induced Depression-like Behaviors via Up-Regulation of 5-HTT and TPH1. *Psychiatry Research*.

[17] Choi G. Y., Kim H. B., Cho J. M. (2023). Umbelliferone Ameliorates Memory Impairment and Enhances Hippocampal Synaptic Plasticity in Scopolamine-Induced Rat Model. *Nutrients*.

[18] Yokosuka A., Mimaki Y., Sashida Y. (2002). Spirostanol Saponins from the Rhizomes of *Tacca Chantrieri* and Their Cytotoxic Activity. *Phytochemistry*.

[19] Armartmuntree N., Kittirat Y., Promraksa B. (2025). Antioxidative and Anticancer Effects of *Tacca Chantrieri* Extract Enhancing Cisplatin Sensitivity in Cholangiocarcinoma Cells. *PLoS One*.

[20] Kittipong K., Chaiyong R., Duangporn A. (2010). Analgesic, Antipyretic and Anti-inflammatory Effects of *Tacca Chantrieri* Andre. *Journal of Medicinal Plants Research*.

[21] Jeeno P., Yadoung S., Yana P., Hongsibsong S. (2023). Phytochemical Profiling and Antioxidant Capacity of Traditional Plants, Northern Thailand. *Plants*.

[22] Kamsrijai U., Thaweethee-Sukjai B., Teerapattarakan N., Wanchai K., Jirarattanawan P. (2023). Neuroprotective Effects of *Tacca Chantrieri* Andre against Lipopolysaccharide-Induced Cognitive Impairment and Neuroinflammation. *Physiology and Pharmacology*.

[23] Yang Y., Gong Q., Wang W. (2020). Neuroprotective and Anti-inflammatory Ditetrahydrofuran-Containing Diarylheptanoids from *Tacca Chantrieri*. *Journal of Natural Products*.

[24] Sandeep Ganesh G., Konduri P., Kolusu A. S. (2023). Neuroprotective Effect of Saroglitazar on Scopolamine-Induced Alzheimer’s in Rats: Insights into the Underlying Mechanisms. *ACS Chemical Neuroscience*.

[25] Shin C. Y., Kim H. S., Cha K. H. (2018). The Effects of Donepezil, an Acetylcholinesterase Inhibitor, on Impaired Learning and Memory in Rodents. *Biomolecules & Therapeutics (Seoul)*.

[26] Thongrong S., Promsrisuk T., Sriraksa N., Surapinit S., Jittiwat J., Kongsui R. (2024). Alleviative Effect of Scopolamine-Induced Memory Deficit via Enhancing Antioxidant and Cholinergic Function in Rats by Pinostrobin from *Boesenbergiarotunda* (L.). *Biomedical Reports*.

[27] Kraeuter A. K., Guest P. C., Sarnyai Z. (2019). The Y-Maze for Assessment of Spatial Working and Reference Memory in Mice. *Methods in Molecular Biology*.

[28] Hawiset T., Sriraksa N., Kongsui R., Kamsrijai U., Wanchai K., Inkaew P. (2023). *Azadirachta Indica* A. Juss. Flower Extract Attenuated Memory Deficit-Induced by Restraint Stress in Male Rats. *Physiology and Pharmacology*.

[29] Zhu X., Wu Y., Pan J. (2021). Neuroinflammation Induction and Alteration of Hippocampal Neurogenesis in Mice Following Developmental Exposure to Gossypol. *International Journal of Neuropsychopharmacology*.

[30] Kim K. Y., Shin K. Y., Chang K. A. (2023). GFAP as a Potential Biomarker for Alzheimer’s Disease: A Systematic Review and Meta-Analysis. *Cells*.

[31] Leng F., Edison P. (2021). Neuroinflammation and Microglial Activation in Alzheimer Disease: Where Do We Go from Here?. *Nature Reviews Neurology*.

[32] Heneka M. T., Carson M. J., Khoury J. E. (2015). Neuroinflammation in Alzheimer’s Disease. *The Lancet Neurology*.

[33] Liu Y., Si Z. Z., Zou C. J. (2023). Targeting Neuroinflammation in Alzheimer’s Disease: From Mechanisms to Clinical Applications. *Neural Regeneration Research*.

[34] Kang I. J., Won M. H., Yan B. (2014). Long-Term Administration of Scopolamine Interferes with Nerve Cell Proliferation, Differentiation and Migration in Adult Mouse Hippocampal Dentate Gyrus, but it Does Not Induce Cell Death. *Neural Regeneration Research*.

[35] Choi J. H., Lee E. B., Jang H. H., Cha Y. S., Park Y. S., Lee S. H. (2021). *Allium Hookeri* Extracts Improve Scopolamine-Induced Cognitive Impairment via Activation of the Cholinergic System and Anti-neuroinflammation in Mice. *Nutrients*.

[36] Khandelwal P. J., Herman A. M., Moussa C. E. H. (2011). Inflammation in the Early Stages of Neurodegenerative Pathology. *Journal of Neuroimmunology*.

[37] Hol E. M., Pekny M. (2015). Glial Fibrillary Acidic Protein (GFAP) and the Astrocyte Intermediate Filament System in Diseases of the Central Nervous System. *Current Opinion in Cell Biology*.

[38] Shibuki K., Gomi H., Chen L. (1996). Deficient Cerebellar Long-Term Depression, Impaired Eyeblink Conditioning, and Normal Motor Coordination in GFAP Mutant Mice. *Neuron*.

[39] Madathil S. K., Carlson S. W., Brelsfoard J. M., Ye P., D’Ercole A. J., Saatman K. E. (2013). Astrocyte-Specific Overexpression of Insulin-like Growth Factor-1 Protects Hippocampal Neurons and Reduces Behavioral Deficits Following Traumatic Brain Injury in Mice. *PLoS One*.

[40] Sofroniew M. V., Vinters H. V. (2010). Astrocytes: Biology and Pathology. *Acta Neuropathologica*.

[41] Purushotham S. S., Buskila Y. (2023). Astrocytic Modulation of Neuronal Signalling. *Frontiers in Network Physiology*.

[42] Ben Haim L., Carrillo-de Sauvage M. A., Ceyzériat K., Escartin C. (2015). Elusive Roles for Reactive Astrocytes in Neurodegenerative Diseases. *Frontiers in Cellular Neuroscience*.

[43] Amaral G. F., Dossa P. D., Viebig L. B. (2016). Astrocytic Expression of GFAP and Serum Levels of IL-1β and TNF-α in Rats Treated with Different Pain Relievers. *Brazilian Journal of Pharmaceutical Sciences*.

[44] Shaftel S. S., Griffin W. S. T., O’Banion M. K. (2008). The Role of Interleukin-1 in Neuroinflammation and Alzheimer Disease: An Evolving Perspective. *Journal of Neuroinflammation*.

[45] Ishijima T., Nakajima K. (2021). Inflammatory Cytokines TNFα, IL-1β, and IL-6 Are Induced in Endotoxin-Stimulated Microglia through Different Signaling Cascades. *Science Progress*.

[46] van Kralingen C., Kho D. T., Costa J., Angel C. E., Graham E. S. (2013). Exposure to Inflammatory Cytokines IL-1β and TNFα Induces Compromise and Death of Astrocytes; Implications for Chronic Neuroinflammation. *PLoS One*.

[47] Glikmann-Johnston Y., Saling M. M., Reutens D. C., Stout J. C. (2015). Hippocampal 5-HT_1A_ Receptor and Spatial Learning and Memory. *Frontiers in Pharmacology*.

[48] Švob Štrac D., Pivac N., Mück-Šeler D. (2016). The Serotonergic System and Cognitive Function. *Translational Neuroscience*.

[49] Azargoonjahromi A. (2024). Serotonin Enhances Neurogenesis Biomarkers, Hippocampal Volumes, and Cognitive Functions in Alzheimer’s Disease. *Molecular Brain*.

[50] Min A. Y., Doo C. N., Son E. J. (2015). N-palmitoyl Serotonin Alleviates Scopolamine-Induced Memory Impairment via Regulation of Cholinergic and Antioxidant Systems, and Expression of BDNF and P-CREB in Mice. *Chemico-Biological Interactions*.

[51] Khan M. I., Karima G., Khan M. Z., Shin J. H., Kim J. D. (2022). Therapeutic Effects of Saponins for the Prevention and Treatment of Cancer by Ameliorating Inflammation and Angiogenesis and Inducing Antioxidant and Apoptotic Effects in Human Cells. *International Journal of Molecular Sciences*.

[52] Liang Y., Yang X., Zhang X. (2016). Antidepressant-Like Effect of the Saponins Part of Ethanol Extract from SHF. *Journal of Ethnopharmacology*.

[53] Samarghandian S., Farkhondeh T., Pourbagher-Shahri A. (2020). Green Tea Catechins Inhibit Microglial Activation Which Prevents the Development of Neurological Disorders. *Neural Regeneration Research*.

[54] Afzal O., Dalhat M. H., Altamimi A. S. A. (2022). Green Tea Catechins Attenuate Neurodegenerative Diseases and Cognitive Deficits. *Molecules*.

[55] Singh S., Sahu K., Kapil L., Singh C., Singh A. (2022). Quercetin Ameliorates Lipopolysaccharide-Induced Neuroinflammation and Oxidative Stress in Adult Zebrafish. *Molecular Biology Reports*.

[56] Dourado N. S., Souza C. D. S., de Almeida M. M. A. (2020). Neuroimmunomodulatory and Neuroprotective Effects of the Flavonoid Apigenin in In Vitro Models of Neuroinflammation Associated with Alzheimer’s Disease. *Frontiers in Aging Neuroscience*.

[57] Li X., Smid S. D., Lin J. (2019). Neuroprotective and Anti-amyloid β Effect and Main Chemical Profiles of White Tea: Comparison against Green, Oolong and Black Tea. *Molecules*.

[58] Dudhgaonkar S., Thyagarajan A., Sliva D. (2009). Suppression of the Inflammatory Response by Triterpenes Isolated from the Mushroom *Ganoderma Lucidum*. *International Immunopharmacology*.

[59] Lyra e Silva N. M., Gonçalves R. A., Pascoal T. A. (2021). Pro-Inflammatory Interleukin-6 Signaling Links Cognitive Impairments and Peripheral Metabolic Alterations in Alzheimer’s Disease. *Translational Psychiatry*.

[60] Iova O. M., Marin G. E., Lazar I. (2023). Nitric Oxide/Nitric Oxide Synthase System in the Pathogenesis of Neurodegenerative Disorders-an Overview. *Antioxidants*.

